# Early-onset parkinsonism as a presenting feature of suspected type 1 Gaucher disease with two pathogenic *GBA1* variants: a case report

**DOI:** 10.3389/fnins.2026.1920271

**Published:** 2026-09-03

**Authors:** Yuqin Sun, Ruihan Ni, Yunli Yu

**Affiliations:** The Affiliated Hospital of Guizhou Medical University, Guiyang, Guizhou, China

**Keywords:** case report, dopamine transporter imaging, early-onset parkinsonism, family segregation, Gaucher disease, *GBA1*, genotype–phenotype correlation, parkinsonism

## Abstract

Parkinson's disease (PD) is a common neurodegenerative disorder, and pathogenic variants in *GBA1* are among its most frequent genetic risk factors. Biallelic pathogenic *GBA1* variants cause Gaucher Disease (GD). In patients with parkinsonism and two detected *GBA1* variants, the absence of biochemical confirmation and allele phasing may make it difficult to determine whether the neurological presentation occurs in the context of GD or represents *GBA1*-associated PD. We report a 35-year-old man with a 7-months history of progressive, asymmetric parkinsonism. Dopamine transporter PET/CT showed reduced bilateral striatal uptake, more pronounced on the right. Abdominal CT showed asplenia, and laboratory eveluation revealed mild thrombocytopenia, marked hyperferritinemia, polyclonal hypergammaglobulinemia, proteinuria, and microscopic hematuria. Whole-exome sequencing followed by Sanger validation identified two heterozygous *GBA1* variants classified as pathogenic: c.1448T>C (p.Leu483Pro; legacy name L444P) and c.1342G>C (p.Asp448His; legacy name D409H). Family testing supported the familial occurrence of both variants but did not establish their allelic phase. Because glucocerebrosidase activity and GD biomarkers were not measured, type 1 Gaucher disease (GD1) remained suspected rather than confirmed. During hospitalization, dopaminergic therapy was associated with clinical improvement, with the OFF-state UPDRS-III score decreasing from 21 at admission to 10 at discharge. The clinical value of this case lies in the convergence of early-onset parkinsonism, remote visceral and hematologic findings, the co-occurrence of p.Leu483Pro and p.Asp448His with unresolved allelic phase, and the familial occurrence of both variants. It underscores the need for biochemical confirmation, *GBA1*-specific phase-resolved testing, and longitudinal multisystem assessment before the diagnosis of GD1 and the associated familial risks can be reliably defined.

## Introduction

Parkinson's disease (PD) is a common neurodegenerative disorder of the central nervous system. Increasing attention has been directed toward the marked clinical heterogeneity of PD, in which genetic factors play a pivotal role in a substantial subset of patients. Pathogenic *GBA1*(encoding the lysosomal enzyme β-glucocerebrosidase, GCase) variants are among the most common known genetic risk factors for Parkinson's disease (PD) and are associated with earlier onset, faster motor progression, and a greater burden of cognitive and other non-motor manifestations (Bloem et al., 2021; Tolosa et al., 2021).

Biallelic pathogenic *GBA1* variants cause Gaucher disease (GD), a lysosomal storage disorder (LSD). Both individuals with GD and heterozygous carriers have an increased risk of PD and related Lewy body disorders, although penetrance and clinical expression vary considerably. Proposed mechanisms include impaired lysosomal and autophagic function, altered lipid homeostasis, mitochondrial dysfunction, neuroinflammation, and a bidirectional relationship between GCase deficiency and α-synuclein accumulation (Vieira and Schapira, 2022; Bougea, 2025; Navarro-Romero et al., 2022).

The determinants of incomplete penetrance and genotype-phenotype variability in *GBA1*-related disorders remain incompletely understood (Bougea, 2025; Lanore et al., 2025). This variability creates a diagnostic challenge. Patients with *GBA1*-related parkinsonism may present with asymmetric bradykinesia, resting tremors, rigidity, and reduced dopamine transporter uptake, closely resembling idiopathic PD (Chinese Society of Parkinson's Disease and Movement Disorders Group of Neurology Branch of Chinese Medical Association, 2020, Jankovic, 2008; Li et al., 2015). Conversely, visceral and hematologic clues suggestive of GD, such as splenomegaly, thrombocytopenia, or a history of splenectomy, may be remote, undocumented, overlooked, or attributed to other conditions. These circumstances may delay recognition of an underlying metabolic disorder, particularly in patients with early-onset parkinsonism.

Here, we report a 35-year-old man with early-onset progressive parkinsonism, a remote history consistent with childhood splenectomy, multisystem abnormalities, and a family history suggestive of GD. Genetic testing identified p.Leu483Pro and p.Asp448His, although their allelic phase remained unresolved. This case shows how early-onset parkinsonism may provide the clinical entry point for recognizing an underlying *GBA1*-related multisystem disorder when remote visceral and hematologic findings have not previously been considered together.

## Case presentation

A 35-year-old man presented to the Department of Neurology in July 2024 with a 7-months history of progressive left-sided parkinsonism. His symptoms began with clumsiness of the left lower limb and dragging of the left leg while walking. Rest tremor subsequently developed in the left lower limb. Three months before admission, he noticed impaired fine motor function and rest tremor in the left upper limb. His bradykinesia progressively worsened, causing difficulty initiating gait, getting out of bed, and dressing.

Non-motor symptoms included low mood, difficulty initiating sleep, and an unintentional weight loss of approximately 4 kg. He denied hyposmia, constipation, hallucinations, and symptoms suggestive of rapid eye movement sleep behavior disorder. No orthostatic hypotension or other autonomic symptoms were identified.

The patient had a vague history of abdominal surgery more than 30 years earlier because of “abdominal discomfort,” but the operative records were unavailable. Examination showed a surgical scar in the left upper abdominal quadrant. His sister had reportedly undergone bone marrow examination for anemia, with findings described by the family as suggestive of Gaucher cells, raising the possibility of a familial lysosomal storage disorder. The original bone marrow examination report was not available for review.

Neurological examination demonstrated masked facies, bilateral bradykinesia (worse on the left), left-sided rest tremor, cogwheel rigidity, and slow alternating movements. Pyramidal signs were present, including brisk deep tendon reflexes in the right lower limb and intermittent clonus. At admission, the modified Hoehn and Yahr stage was 1.5, and the OFF-state Unified Parkinson's Disease Rating Scale part III (UPDRS-III) score was 21. Cognitive screening showed an MMSE score of 29/30, a MoCA score of 28/30, and a Clock Drawing Test score of 3/4.

Laboratory testing revealed mild thrombocytopenia (114 × 10^9^/L), marked hyperferritinemia (2,180 ng/mL), and polyclonal hypergammaglobulinemia (IgA 8.71 g/L; IgM 3.80 g/L). Autoantibodies testing showed an antinuclear antibody titer of 1:100, weakly positive anti–PM-Scl antibody, and positive anti–SS-A52 antibody. Urinalysis showed persistent proteinuria (2,822.66 mg/24 h) and microscopic hematuria. Serum creatinine was normal, and the estimated glomerular filtration rate was 89.15 mL/min/1.73 m^2^. Renal ultrasonography showed no significant structural abnormality. Serum ceruloplasmin was normal (339.4 mg/L). Other investigations for infectious, autoimmune, endocrine, and neoplastic causes did not reveal additional significant abnormalities. Electrocardiography showed sinus rhythm without abnormalities.

**Figure 1:**
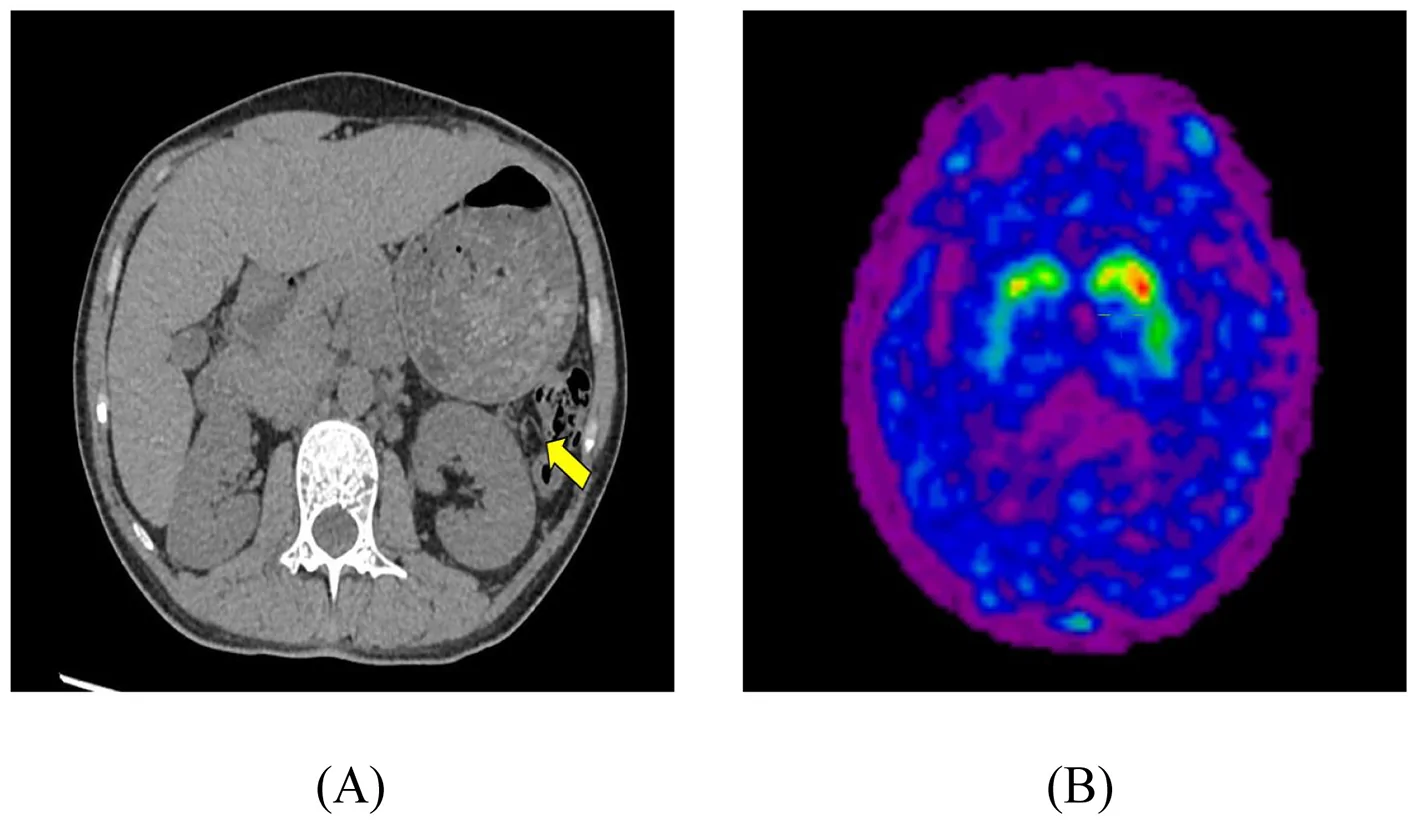
Multimodal imaging findings. **(A)** Abdominal CT showing absence of the spleen (arrow). **(B)** Dopamine transporter PET/CT demonstrating reduced radiotracer uptake in the bilateral putamen, more pronounced on the right.

Abdominal CT demonstrated altered hepatic morphology, portal vein dilatation, and non-visualization of the spleen, consistent with probable previous splenectomy (Figure 1A). Transient elastography indicated increased liver stiffness (10.1 kPa) with mild hepatic steatosis. Brain MRI, including susceptibility-weighted imaging showed no significant intracranial abnormalities. Dopamine transporter PET/CT demonstrated markedly reduced bilateral striatal uptake, which was more pronounced on the right (Figure 1B).

Given the early onset parkinsonism, asplenia, mild thrombocytopenia, hepatobiliary abnormalities, and suggestive family history, genetic evaluation was prompted. Whole-exome sequencing was performed using peripheral-blood genomic DNA. Exonic regions and adjacent splice sites were captured using the Roche KAPA HyperExome panel, followed by high-throughput sequencing. Two heterozygous *GBA1* variants were identified: NM_001005741.2:c.1342G>C (p.Asp448His; legacy name D409H) and NM_001005741.2:c.1448T>C (p.Leu483Pro; legacy name L444P). Both were classified as pathogenic by the testing laboratory and confirmed in the proband by Sanger sequencing (Table 1). No phenotype-associated pathogenic or likely pathogenic chromosomal copy-number variants were detected.

**Table 1:** *GBA1* variants identified in the proband.

Gene	Transcript	Variant (cDNA)	Protein change	Exon	Zygosity	ACMG classification
*GBA1*	NM-001005741.2	c.1342G>C	p.Asp448His	Exon 10	Heterozygous	Pathogenic
*GBA1*	NM-001005741.2	c.1448T>C	p.Leu483Pro	Exon 11	Heterozygous	Pathogenic

**Figure 2:**
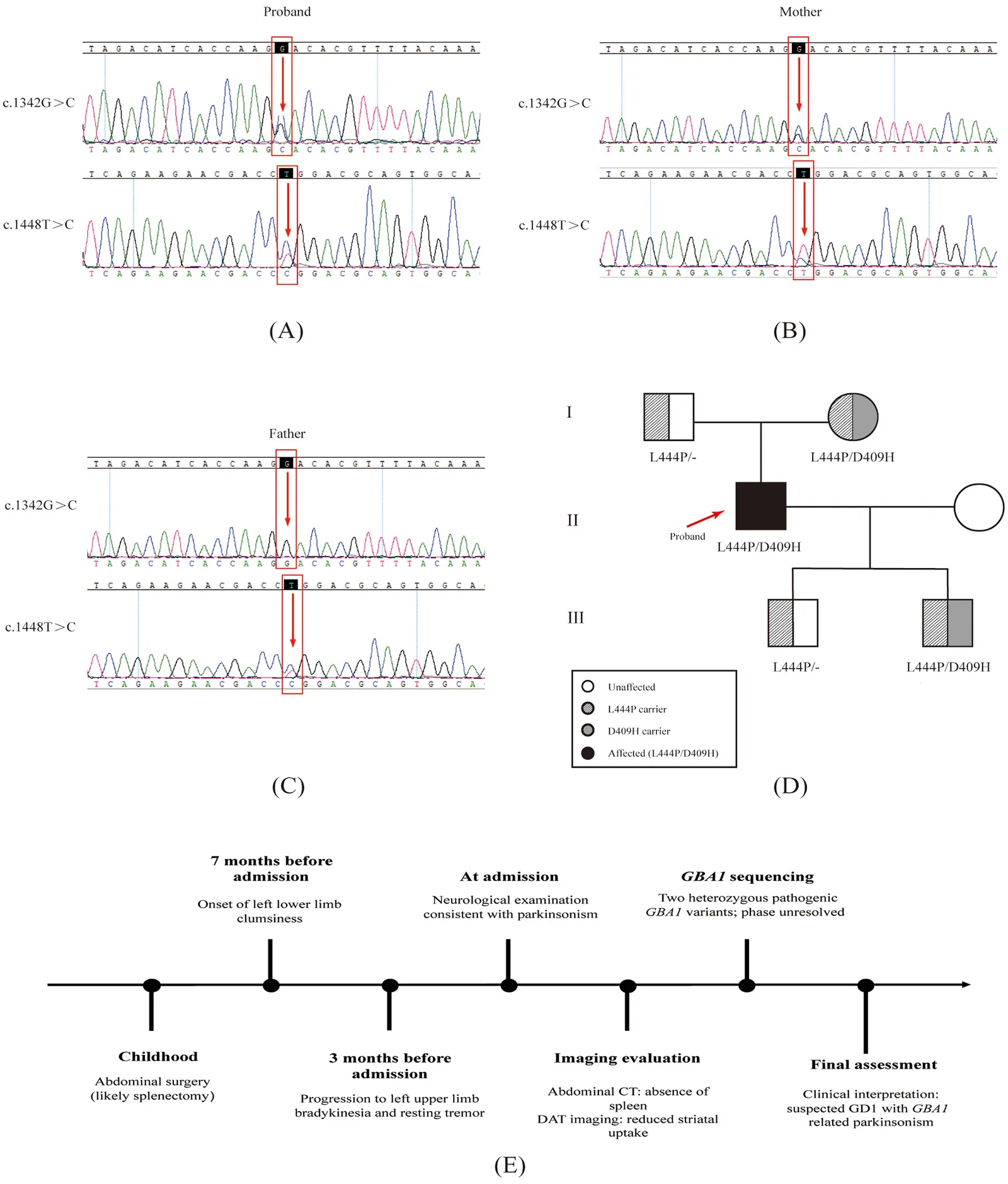
Genetic findings and family segregation analysis of *GBA1* variants. **(A–C)** Sanger sequencing results showing the identified *GBA1* variants in the proband **(A)**, the mother **(B)**, and the father **(C)**. The proband carried two pathogenic *GBA1* variants, c.1448T>C (p.Leu483Pro) and c.1342G>C (p.Asp448His). The mother carried both variants, whereas the father carried c.1448T>C (p.Leu483Pro) alone. **(D)** Pedigree of the family showing the segregation pattern of *GBA1* variants. The proband and younger son carried both *GBA1* variants, while the father and elder son carried p.Leu483Pro alone. The phase relationship of the two variants in the proband was not directly determined. **(E)** Clinical timeline summarizing the major clinical events, diagnostic evaluations, genetic testing, and final clinical interpretation.

Family testing showed that the father carried p.Leu483Pro, whereas the mother carried both variants. The elder son carried p.Leu483Pro, and the younger son carried both variants according to the available family report (Figures 2A–D). However, the maternal variants were not phased, and the proband's spouse was not tested. The parental origin of p.Leu483Pro in the proband therefore remained undetermined. The available results did not establish whether the two variants were in cis or in trans.

Dopaminergic therapy improved the patient's motor symptoms. At discharge, the OFF-state UPDRS-III score had decreased from 21 to 10. Both assessments were performed in the OFF state. He was discharged on pramipexole hydrochloride 0.125 mg three times daily and levodopa/benserazide 62.5 mg three times daily. Enzyme replacement therapy was not initiated because GD had not been biochemically confirmed. Further hepatologic, hematologic, and nephrologic evaluation was recommended for the marked hyperferritinemia, proteinuria, and hepatic abnormalities. The patient was subsequently lost to follow-up; therefore, his status at discharge was the last available clinical assessment. The key clinical events are summarized in Figure 2E.

## Discussion

This case illustrates a diagnostic pathway in which early-onset parkinsonism prompted recognition of a probable *GBA1*-related disorder. The patient initially presented with asymmetric rest tremor, bradykinesia, rigidity, and reduced DAT uptake, which were clinically consistent with PD. However, onset at 35 years of age, relatively rapid progression, pyramidal signs, remote abdominal surgery, asplenia, thrombocytopenia, hepatobiliary abnormalities, and a suggestive family history broadened the differential diagnosis (Bloem et al., 2021; Tolosa et al., 2021). Genetic testing identified two pathogenic *GBA1* variants. Nevertheless, the absence of biochemical confirmation and resolved allelic phase precluded a definitive diagnosis of GD1. Consequently, the case is best interpreted as suspected GD1 with *GBA1*-related parkinsonism.

Although GD1 is conventionally classified as non-neuronopathic (Grabowski et al., 2015; Hruska, 2008), patients with GD1 and heterozygous *GBA1* variant carriers have an increased risk of PD and related parkinsonism (Bultron et al., 2010). GD-associated parkinsonism may closely resemble idiopathic PD, making recognition of the underlying metabolic disorder challenging (Collins et al., 2018). In the present case, the remote visceral and hematologic findings provided important evidence that the parkinsonian syndrome might not represent isolated PD. This emphasizes the value of reviewing remote systemic and surgical histories in patients with early-onset parkinsonism.

Previous series have shown that parkinsonism associated with GD may begins earlier than sporadic PD and may show asymmetric tremor, rigidity, akinesia, variable levodopa responsiveness, and heterogeneous cognitive outcomes (Collins et al., 2018; Tayebi et al., 2003). The present case does not establish a new general association between *GBA1* and parkinsonism. Its clinical value lies in the convergence of very early parkinsonism, long-overlooked systemic findings, two pathogenic *GBA1* variants, and an informative but unresolved family segregation pattern.

The p.Leu483Pro variant is common among Chinese patients with GD. Homozygosity or its occurrence within a complex allele has been associated with neuronopathic phenotypes (Li et al., 2015; Red Blood Cell Disease (Anemia) Group CMA, 2020). By contrast, p.Asp448His is less frequently reported in Asian populations (Vieira et al., 2024). Homozygous p.Asp448His has been associated with a distinct phenotype involving oculomotor abnormalities and cardiovascular calcification (Park et al., 2003; Pasmanik-Chor et al., 1996). The two variants were detected together, but their allelic phase and the presence of a complex or recombinant allele were not determined. The apparent discrepancy between variants associated with severe phenotypes and the patient's relatively limited neurological findings should therefore be interpreted cautiously. It does not establish a new genotype–phenotype relationship.

Family testing demonstrated the familial occurrence of both variants but did not resolve their allelic configuration. The father carried p.Leu483Pro, whereas the mother carried both p.Leu483Pro and p.Asp448His. Moreover, both parents carried p.Leu483Pro, preventing determination of its parental origin in the proband. If the two variants were assumed to be located on the same maternal allele (cis), both variants in the proband would have been inherited from the mother. However, p.Leu483Pro and p.Asp448His are located only approximately 106 bp apart within the coding region, and meiotic recombination between such closely located variants is expected to be rare. Therefore, variants located on the same allele would generally be expected to cosegregate as a linked unit in offspring. In this family, the elder son carried p.Leu483Pro alone, whereas the younger son carried both variants, making a cis configuration less consistent with the observed segregation pattern and favoring a trans configuration. Thus, the two variants in the proband probably originated from the paternal and maternal alleles, respectively. Nevertheless, the proband's spouse was not tested, which also limited interpretation of the variants detected in their sons. This interpretation remains provisional.

Genetic interpretation was further limited by the technical complexity of *GBA1* analysis. The highly homologous pseudogene *GBAP1* can interfere with short-read sequencing and complicate the detection of recombinant alleles, gene conversion, and copy-number variation (Hruska, 2008; Toffoli et al., 2022). Although Sanger sequencing confirmed both variants in the proband, it did not determine their phase or exclude all complex *GBA1* alleles. *GBA1*-specific phase-resolved testing would therefore be required before biallelic disease or familial recurrence risks could be established.

Previous studies have demonstrated that *GBA1*-associated parkinsonism can be presented as isolated parkinsonism or occur in the context of systemic Gaucher disease. To better characterize the clinical and genetic features of the present case, we compared the neurological manifestations, systemic involvement, and *GBA1* genotypes with previously reported cases (Table 2) (Tayebi et al., 2001; Le Peillet et al., 2018; Machaczka et al., 2012). However, the comparison is descriptive because biochemical confirmation, phase analysis, and clinical assessments were not reported consistently across cases. The present case should therefore be viewed as an additional diagnostic example rather than evidence for a distinct clinical subtype.

**Table 2:** Clinical and genetic comparison of the present case with previously reported cases of *GBA1*-associated parkinsonism.

Reference/ case	Present case	(Tayebi et al. 2001)	(Le Peillet et al. 2018)	(Machaczka et al. 2012)
Age at parkinsonism onset	35	42	62	39
*GBA1* variants	p.Leu483Pro (L444P)/p.Asp448His (D409H)	p.Leu483Pro (L444P)/p.Asp448His (D409H)	p.Asn409Ser (N370S)/p.Asp448His (D409H)	c.115+1G>A (IVS2+1)/c.1226A>G (N370S)
Variant phase confirmation	Unresolved (cis/trans phase not determined)	Not clearly established	Not reported	Not reported
GD confirmation	Suspected GD1 (biochemical confirmation unavailable)	Confirmed GD1	Confirmed GD1	Confirmed GD1
Parkinsonism phenotype	Early-onset asymmetric parkinsonism	Tremor-onset parkinsonism with progressive rigidity, bradykinesia and gait impairment; atypical progression	Parkinson disease with asymmetric motor symptoms; dopaminergic response	Early-onset asymmetric parkinsonism preceding GD manifestation; levodopa-responsive initially
Cognitive involvement	Mild/no impairment	Cognitive decline during disease progression	Not reported	Mild/no major impairment (MMSE 27/30 reported before pallidotomy)
Hematological findings	Thrombocytopenia	History of bleeding tendency; hematological abnormalities not prominent at report	Thrombocytopenia, anemia	Thrombocytopenia
Hepatic/splenic involvement	History of splenectomy; hepatobiliary abnormalities	Splenomegaly requiring splenectomy; hepatomegaly	Hepatosplenomegaly	Splenomegaly (normalized after ERT); no major liver involvement emphasized
Other systemic findings	Proteinuria, hematuria, hypergammaglobulinemia, and autoantibodies	Oculomotor abnormalities; myoclonus/fasciculations	Bullous pemphigoid; monoclonal gammopathy	RBD-like symptoms; systemic response to ERT but persistent neurological progression

Compared with previously reported cases, the present patient exhibited not only typical parkinsonian manifestations but also multiple systemic abnormalities. The asplenia, thrombocytopenia, altered hepatic morphology, portal vein dilatation, and increased liver stiffness were compatible with systemic involvement. Notably, this patient also presented with renal abnormalities, including significant proteinuria and microscopic hematuria, while renal function remained relatively preserved. Although renal involvement is not a common manifestation of GD and has been reported more frequently in pediatric patients, previous studies have described renal involvement in some GD patients, membranoproliferative glomerulonephritis and Gaucher cell infiltration within glomerular capillaries (Stirnemann et al., 2017; Liang et al., 2023). Therefore, longitudinal renal monitoring and further renal evaluation, including renal biopsy when clinically appropriate, are important considerations for this patient. In addition, the patient exhibited marked hyperferritinemia and hypergammaglobulinemia. Although these abnormalities are not typical diagnostic features of GD, they may have potential associations with GD, considering that GD is a lysosomal storage disorder characterized by abnormal macrophage activation and chronic immune dysregulation (Le Peillet et al., 2018; Stirnemann et al., 2017; Soroglu and Berkay, 2024). Previous studies have suggested that autoantibody positivity is more frequent in patients with GD than in healthy individuals; however, these autoantibodies have not been associated with an increased prevalence of clinically active autoimmune diseases (Serratrice et al., 2016). Therefore, whether the weakly positive autoantibodies observed in this patient reflect GD-related immune dysregulation or merely represent nonspecific immune abnormalities remains uncertain.

Cardiac involvement was not systematically assessed because echocardiography was not performed. Although the patient had no cardiovascular symptoms or electrocardiographic abnormalities, subclinical structural or valvular involvement could not be excluded. This limitation is relevant because cardiac calcification has been described in specific p.Asp448His-associated genotypes, particularly homozygous cases (Hruska, 2008; Pasmanik-Chor et al., 1996; Tayebi et al., 2001). Additional cardiac assessment would have been appropriate if follow-up had been available.

However, because the patient did not complete further disease-specific investigations, including glucocerebrosidase activity measurement, chitotriosidase and GlcSph biomarker testing, as well as additional assessments to evaluate possible GD involvement, definitive classification remains challenging (Vieira et al., 2024; Toffoli et al., 2022). Therefore, the possibility of *GBA1*-associated early-onset Parkinson's disease (for example, occurring in a *GBA1* carrier or complex-allele state) accompanied by independent hepatic, hematological, or renal disorders, or another multisystem disease, cannot be excluded. Furthermore, due to the lack of formal phasing and analysis of *GBA1*/GBAP1 recombinant alleles and copy-number variations, the current genetic interpretation should be considered with caution (Vieira et al., 2024; Toffoli et al., 2022).

Importantly, it should be emphasized that marked hyperferritinemia, hypergammaglobulinemia, positive autoantibodies, proteinuria, and hematuria should not be directly attributed to GD without further evidence. Differential diagnoses should include inflammatory or autoimmune disorders, chronic liver injury, metabolic or iron-overload conditions, and primary renal diseases. During hospitalization, multidisciplinary consultations, including nephrology, endocrinology, and infectious disease specialists, were obtained because of the multisystem abnormalities; however, no definitive alternative diagnosis was established, and these evaluations could not completely exclude other potential explanations. Therefore, continued follow-up and specialist evaluation remain important (Levin et al., 2023). Dopaminergic responsiveness supports the diagnosis of a dopaminergic-responsive parkinsonian syndrome and guides symptomatic treatment, but does not determine the underlying systemic diagnosis.

## Conclusion

This case shows how suspected GD1 may be overlooked in a young patient presenting with parkinsonism when visceral and hematologic clues are remote. The combined clinical findings and two pathogenic *GBA1* variants support suspected GD1 with *GBA1*-related parkinsonism, but do not establish biochemically confirmed GD1 or a new genotype–phenotype relationship.

The case's principal contribution is diagnostic: it brings together early-onset parkinsonism, long-overlooked systemic clues, an uncommon p.Leu483Pro/p.Asp448His combination, and an informative but unresolved family segregation pattern. Enzyme and biomarker testing, phase-resolved *GBA1* analysis, systematic evaluation of relatives, and long-term multidisciplinary follow-up remain necessary to define disease status and familial risk.

## Data Availability

The data supporting the findings of this study are available from the corresponding author upon reasonable request.
